# The efficacy and safety of disitamab vedotin combined with immune checkpoint inhibitors in metastatic upper tract urothelial carcinoma: a multicenter real-world study

**DOI:** 10.1007/s00262-025-04154-5

**Published:** 2025-09-13

**Authors:** Cheoklong Ng, Taile Jing, Shicheng Yu, Jianjun Ye, Shun Zhang, Zhankui Jia, Qi Tang, Xiaoyi Hu, Minfeng Chen, Weiping Huang, Jitao Wu, Hai Bi, Zejun Yan, Haibo Shen, Wei Xiong, Zheng Liu, Jun Xiao, Tao Zhang, Xuedong Wei, Hailong Hu, Qing Yang, Yichu Yuan, Zhiyang Huang, Wei Xue, Yige Bao, Guoqing Ding, Dan Xia, Jiwei Huang

**Affiliations:** 1 Department of Urology, Renji Hospital, Shanghai Jiao Tong University School of Medicine, Shanghai, 200127 China; 2 The First Affiliated Hospital, School of Medcine, Zhejiang University, #79 Qingchun Rd., Hangzhou, 310003 Zhejiang Province People’s Republic of China; 3 Department of Urology, Sir Run Run Shaw Hospital, School of Medicine Zhejiang University, No.3 East Qingchun Road, Hangzhou, People’s Republic of China; 4 Department of Urology, West China Hospital Sichuan University, Chengdu, 610041 China; 5 Department of Urology, Affiliated Drum Tower Hospital Medical School of Nanjing University, Nanjing, People’s Republic of China; 6 The First Affiliated Hospital of Zhengzhou University, No. 1 Jianshe East Road, Zhengzhou, China; 7 Department of Urology, Peking University First Hospital, Peking University, National Urological Cancer Center, Beijing, China; 8 Department of Urology, Zhongshan Hospital, Fudan University, No. 180, Fenglin Road, Shanghai, China; 9 Department of Urology, Xiangya Hospital, Central South University, Xiangya Street, Changsha, 41008 Hunan China; 10 Department of Urology, the First Affiliated Hospital of Wenzhou Medical University, Wenzhou, Zhejiang People’s Republic of China; 11 Department of Urology, the Affiliated Yantai Yuhuangding Hospital of Qingdao University, Yantai, Shandong China; 12 Department of Urology, Shanghai General Hospital, Shanghai Jiao Tong University School of Medicine, No. 100 Haining Road, Hongkou District, Shanghai, China; 13 Department of Urology, the First Affiliated Hospital of Ningbo University, Ningbo, 315010 China; 14 Department of Urology, Xinhua Hospital Affiliated to Shanghai Jiao Tong University School of Medicine, 1665 Kongjiang Road, Shanghai, China; 15 Department of Urology, Sichuan Academy of Medical Sciences and Sichuan Provincial People’s Hospital, Chengdu, China; 16 Department of Urology, Tongji Hospital, Tongji Medical College, Huazhong University of Science and Technology, Wuhan, 430030 China; 17 Department of Urology, The First Affiliated Hospital of USTC, Division of Life Sciences and Medicine, University of Science and Technology of China, No 17, Lujiang Road, Luyang District, Hefei, 230001 Anhui China; 18 Department of Urology, The Second Affiliated Hospital of Anhui Medical University, Hefei, 230601 Anhui People’s Republic of China; 19 The First Affiliated Hospital of Soochow University, Pinghai Rd. 899#, Suzhou, 21500 People’s Republic of China; 20 Department of Urology, the Second Hospital of Tianjin Medical University, Tianjin, People’s Republic of China; 21 Department of Urology, Shanghai Ninth People’s Hospital, Shanghai Jiaotong University School of Medicine, Shanghai, 200011 People’s Republic of China; 22 Department of Urology, The Second Affiliated Hospital, School of Medicine, Zhejiang University, No.88 Jiefang Road, Hangzhou, 310009 Zhejiang People’s Republic of China; 23 Department of Urology, Quanzhou First Hospital affiliated to Fujian Medical University, Quanzhou, People’s Republic of China

**Keywords:** Disitamab vedotin, Immune checkpoint inhibitors, Upper tract urothelial carcinoma, Targeted therapy, Immunotherapy

## Abstract

**Background:**

Disitamab vedotin (DV, RC48-ADC) has shown promising efficacy and manageable safety as monotherapy or combined with immune checkpoint inhibitors (ICIs) in metastatic urothelial carcinoma (mUC) patients progressing after systemic chemotherapy. This study evaluates the efficacy and safety of RC48-ADC plus ICIs in metastatic upper tract urothelial carcinoma (mUTUC) in a real-world setting.

**Methods:**

This retrospective, multicenter study included 198 mUTUC patients treated with RC48-ADC plus ICI at 23 clinical centers between June 2021 and December 2023. Data were analyzed from July to September 2024. Primary endpoint: objective response rate (ORR). Secondary endpoints: progression-free survival (PFS), overall survival (OS), duration of response (DoR), time to objective response, and treatment-related adverse events (TRAEs).

**Result:**

Among the patients, 122 (61.6%) received combination therapy as first-line treatment, while 76 (38.4%) as second-line or beyond. ORR was 58.6% (95% CI: 51.4–65.5), with a median PFS of 13.0 months; median OS was not reached. Subgroup analyses showed consistent ORR across all subgroups. Adverse events (AEs) occurred in 79.8% of patients, with grade 3–4 AEs in 19.7%. Common AEs included neuropathy (40.4%), fatigue (26.8%), alopecia (25.3%), and rash (21.7%). The main limitations of this study include its retrospective design and the population of the study only include patients from China, leading to potential selection bias.

**Conclusions:**

RC48-ADC combined with ICIs demonstrated robust antitumor activity and a favorable safety profile in real-world mUTUC, particularly in selected patient groups. Further studies are needed to explore its potential in this high-risk population.

**Supplementary Information:**

The online version contains supplementary material available at 10.1007/s00262-025-04154-5.

## Introduction

Urothelial carcinoma (UC) is a type of cancer that arises from urothelial cells, occurring in both the upper urinary tract (renal pelvis and ureter) and lower urinary tract (bladder). It ranks as the fourth most commonly diagnosed cancer worldwide [1]. Upper tract UC (UTUC) accounted for 5–10% of all UC cases. Patients with metastatic UTUC have a poor prognosis. Cisplatin-based chemotherapy has been the first-line standard therapy for mUC patients for a long time [2]. However, nearly half of patients are ineligible for cisplatin-based chemotherapy due to factors including renal impairment, neuropathy, and hearing loss [3, 4]. Immune checkpoint inhibitors (ICIs) have become the standard second-line treatment for patients with UC; however, only about 25% of patients have a response to ICIs [5, 6]. Therefore, identifying effective treatment options for metastatic UC is essential.

In recent years, several studies have reported that antibody–drug conjugates (ADCs) have the potential to reduce systemic toxicity and improves efficacy of immunotherapy through regulating the immune system [7, 8]. As a result, ADCs are being increasingly used in combination with other therapeutic agents [9].

Disitamab vedotin (DV, RC48-ADC) is an innovative ADC that links hertuzumab (a novel anti-HER-2 monoclonal antibody) with monomethyl auristatin E (MMAE) via a cleavable linker. Clinical trials of RC48-C005 and RC48-C009 showed promising efficacy and manageable safety in patients with mUC who had progressed after previous systemic chemotherapy, leading to the approval of DV in China in June 2021 for platinum-refractory patients with metastatic UC [10]. In addition, the combination therapy of RC48-ADC and toripalimab has shown promising efficacy and a manageable safety profile in patients with mUC, as demonstrated by RC48-C014 study [11]. The objective of this multicenter, retrospective study is to examine the efficacy and safety of RC48-ADC plus ICIs therapy in patients with mUTUC in the real-world setting.

## Methods

This retrospective, multicenter, real-world study enrolled 268 patients treated across 23 clinical centers of the Chinese Urological Doctors Association-UTUC collaborative group (CUDA-UTUCCG). Inclusion criteria are as follows: (1) Diagnosed mUTUC through imaging test (CT scan, MRI, or PET-CT) and biopsy; (2) Underwent at least 1 cycle of RC48-ADC in combination with ICIs from June 1, 2021, to December 30, 2023. Per protocol, no comparator data (e.g., chemotherapy or RC48-ADC monotherapy recipients) were collected; and (3) At least one measurable metastatic lesions per RECIST v1.1 and one time of treatment evaluation. All patients underwent regular survival follow-up and assessment of treatment efficacy and AEs at intervals of 6 to 12 weeks. Patients were considered evaluable only if they completed ≥ 2 treatment cycles (≥ 6 weeks) and underwent ≥ 1 post-baseline radiographic assessment. This ensured adequate treatment exposure for response evaluation per RECIST v1.1 guidelines. Exclusion criteria were incomplete treatment records, mainly about the treatment evaluation and adverse event records. The treatments were administered as an intravenous infusion every 3 weeks until disease progression, unacceptable toxicity, death, or withdrawal of consent. The dose of RC48-ADC and ICIs would be modified or interrupted based on patients’ adverse events (AEs). HER-2 status was determined locally at each participating institution using immunohistochemistry (IHC) performed in accordance with standardized ASCO/CAP guidelines. Data cutoff was June 30, 2024. Data analysis was conducted from July 2024 to September 2024. This study adhered to the Strengthening the Reporting of Observational Studies in Epidemiology (STROBE) reporting guideline.

### Study endpoints and assessments

The primary endpoint of this study was objective response rate (ORR), which was evaluated in all eligible patients. The ORR is defined as the percentage of patients who achieve at least one response evaluation showing either partial response (PR) or complete response (CR) to treatment. Assessment of response was conducted according to the Response Evaluation Criteria in Solid Tumors version 1.1 (RECIST v1.1) by treating physicians at each participating center. Secondary endpoints comprised progression-free survival (PFS), overall survival (OS), duration of response (DoR), time to objective response, and treatment-related adverse events (TRAEs) of patients. TRAEs occurring in patients were monitored using the Common Terminology Criteria for Adverse Events version 5.0 (CTCAE v5.0).

### Statistical analysis

Statistical analysis was performed using IBM SPSS statistics 27.0 and R 4.4.0 from July 2024 to September 2024. A two-sided *P* < 0.05 was considered as statistically significant. The 95% confidence interval (95%CI) was calculated using the Clopper-Pearson method. OS was defined as duration from treatment start to death or censored at last follow-up. PFS was defined as the time from the start of treatment to the first evaluation of progressive disease (PD). Patients without progression/death events were right-censored, at the timeline of: (1) Last verified contact date for OS and (2) Last radiologically confirmed disease assessment date for PFS. The Kaplan–Meier method was utilized to estimate PFS in subgroups of interest (HER-2 status, treatment line, best overall response, and metastatic status of patients). The cox regression analysis was employed to compare the efficacy across specified subgroups of patients with mUTUC, including age, gender, PD-L1 expression, HER-2 expression, Bajorin risk score, primary tumor location, metastatic status, eGFR, and line of therapy.

To address missing data in key variables (primarily HER-2 and PD-L1 status of unknown, with missing rates of 7.1% and 22.2%, respectively), we employed multiple imputation (MI) using the MICE package in R (v4.4.0). The imputation protocol included: (1) Targeted Variables: HER-2 IHC status (dichotomized: 0/1 + vs. 2 + /3 +); PD-L1 expression (dichotomized: < 1% vs. ≥ 1%); (2) Imputation Model: Predictive mean matching (PMM) for continuous variables; logistic regression for binary variables; (3) Execution: 50 iterations *5 imputed datasets; convergence verification through automated missingness rate assessment; best dataset selection according to minimal combined missing rate criterion; (4) Downstream Analysis: The related Kaplan–Meier curves generated from the minimal missingness imputed dataset; Cox models applied directly to selected dataset.

## Result

### Patient characteristics

**Fig. 1:**
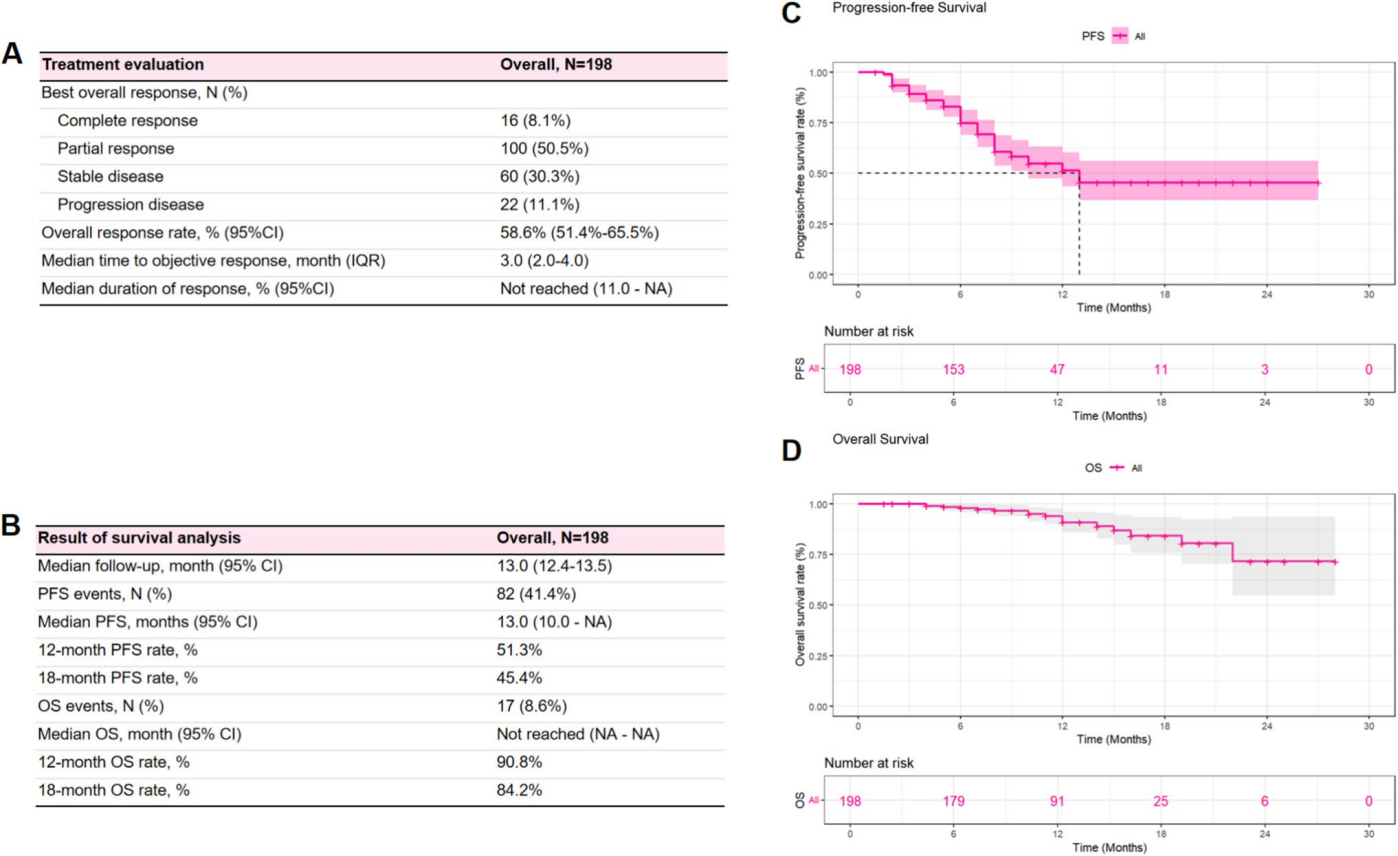
Treatment evaluation and survival analysis of patients

A total of 268 patients from 23 clinical centers were included in this retrospective study. As of the data cutoff date (June 30, 2024), 198 patients were included in the final statistical analysis (S- Fig. 1). There are 70 patients who were excluded from the final analysis because of: (1) incomplete imaging data at baseline and/or after treatment (*n* = 35), leading to the difficulties in treatment evaluation; and (2) duration of treatment was insufficient for at least one treatment evaluation (< 6 weeks or 2 cycle of treatment) (n = 35). Patients received RC48-ADC at a dose of 60 mg (7.6%), 120 mg (90.9%), 180 mg (1.0%), or 240 mg (0.5%), in combination with specific ICIs, primarily toripalimab (240 mg) (52.5%), tislelizumab (200 mg) (33.8%), and pembrolizumab (200 mg) (6.6%). The baseline characteristics of patients are summarized in Table 1. The median age was 68.0 years (range: 38.0–99.0). Of the 198 patients, 133 (67.2%) patients were male, 139 (70.2%) patients had their primary tumor located exclusively in renal pelvis and ureter, while 59 (29.8%) have tumors located in both renal pelvis and ureter. Most patients had the eGFR of more than 60 mL/min/1.73m^2^ (61.1%), an ECOG PS of 1 (78.3%), and a Bajorin risk score of 1 (46.0%) or 2 (50.0%). Visceral metastases were presented in 47.5% of patients, and the main sites of visceral metastasis were liver (15.1%) and lung (23.2%). Bone metastasis occurred in 22.7% of patients. One hundred and twenty-two (61.6%) of patients received combination therapy of RC48-ADC and ICI as first-line therapy, with the main ICIs used being toripalimab (52.5%), tislelizumab (33.8%), and pembrolizumab (6.6%). In terms of immunophenotype, 72.7% of patients had HER-2 expression of IHC (2 + or 3 +), and 36.4% had high PD-L1 expression (≥ 1% or CPS ≥ 10).

**Table 1:** Baseline characteristics of patients

Characteristic	N (%)
Overall	198 (100%)
Median age, year (range)	68.0 (38.0–99.0)
Gender	
Male	133 (67.2%)
Female	65 (32.8%)
Primary tumor lesion(s)	
Renal pelvis	77 (38.9%)
Ureter	62 (31.3%)
Both	59 (29.8%)
eGFR, mL/min/1.73m2	
	10 (5.1%)
≥ 30	81 (40.9%)
≥ 60	107 (54.0%)
ECOG PS	
0	24 (12.1%)
1	155 (78.3%)
≥ 2	19 (9.6%)
Bajorin risk score	
0	75 (37.9%)
1	111 (56.0%)
2	12 (6.1%)
Pathology	
Pure urothelial	153 (77.3%)
Mixed urothelial	45 (22.7%)
Grade	
High	180 (90.9%)
Low	7 (3.5%)
Unknown	11 (5.6%)
With lymphovascular invasion	
Yes	43 (21.7%)
No	144 (72.7%)
Unknown	11 (5.6%)
Metastasis	
Only lymph node metastasis	80 (40.4%)
Bone Metastasis	45 (22.7%)
Visceral metastasis	94 (4.75%)
Liver	30 (15.1%)
Lung	46 (23.2%)
Line of therapy	
First line	122 (61.6%)
Second line	65 (32.8%)
Third line or beyond	11 (5.6%)
Type of immune checkpoint inhibitor	
Toripalimab	104 (52.5%)
Tislelizumab	67 (33.8%)
Pembrolizumab	13 (6.6%)
Others	14 (7.1%)
HER-2 expression	
HER-2 (IHC 2 + or 3 +)	144 (72.7%)
HER-2 (IHC 0 or 1 +)	40 (20.2%)
HER-2 unknown	14 (7.1%)
PD-L1 expression	
PD-L1 (≥ 1% or CPS ≥ 10)	72 (36.4%)
PD-L1 (< 1% or CPS < 10)	82 (41.4%)
PD-L1 Unknown	44 (22.2%)

### Efficacy

**Fig. 2:**
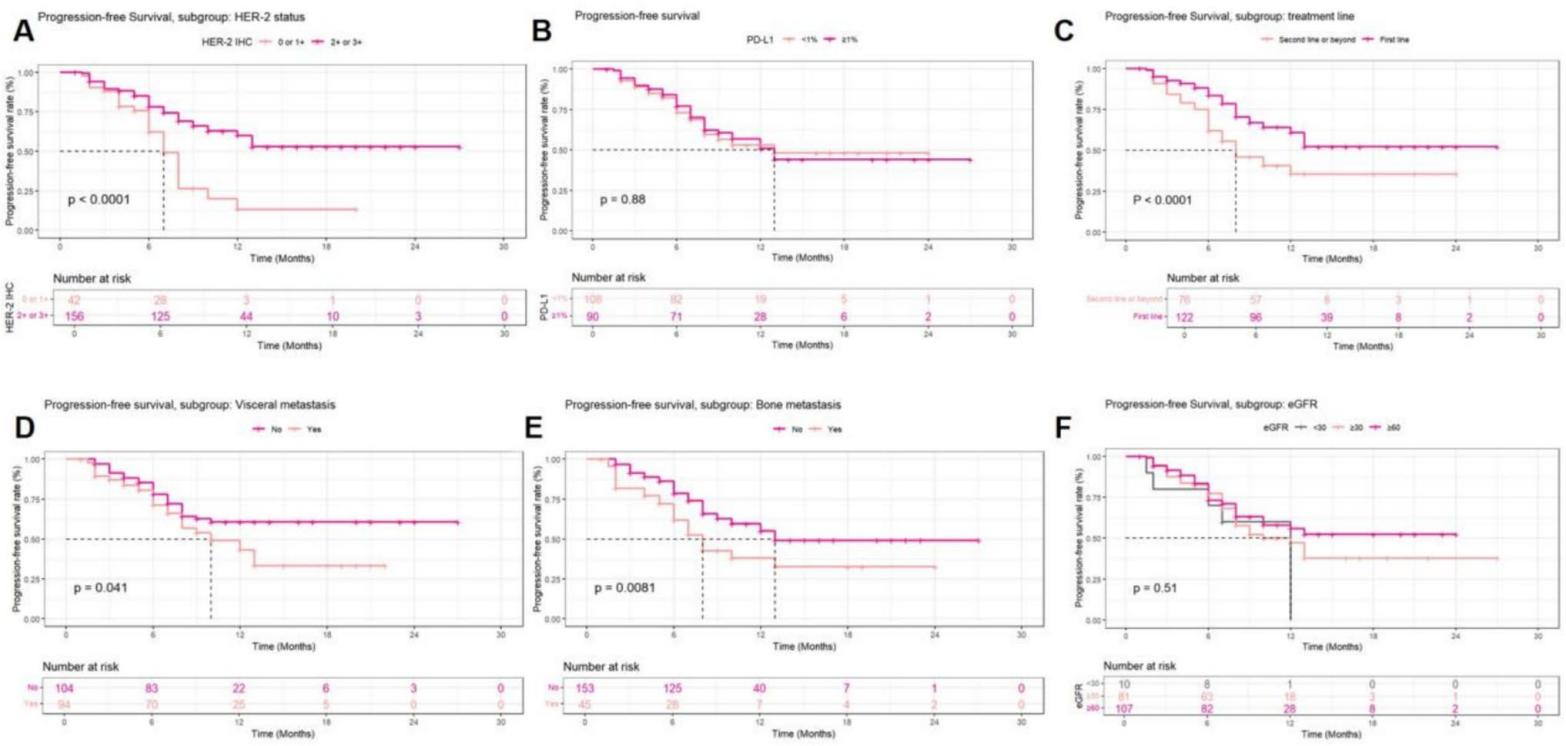
Progression-free survival of patients in different subgroups

**Fig. 3:**
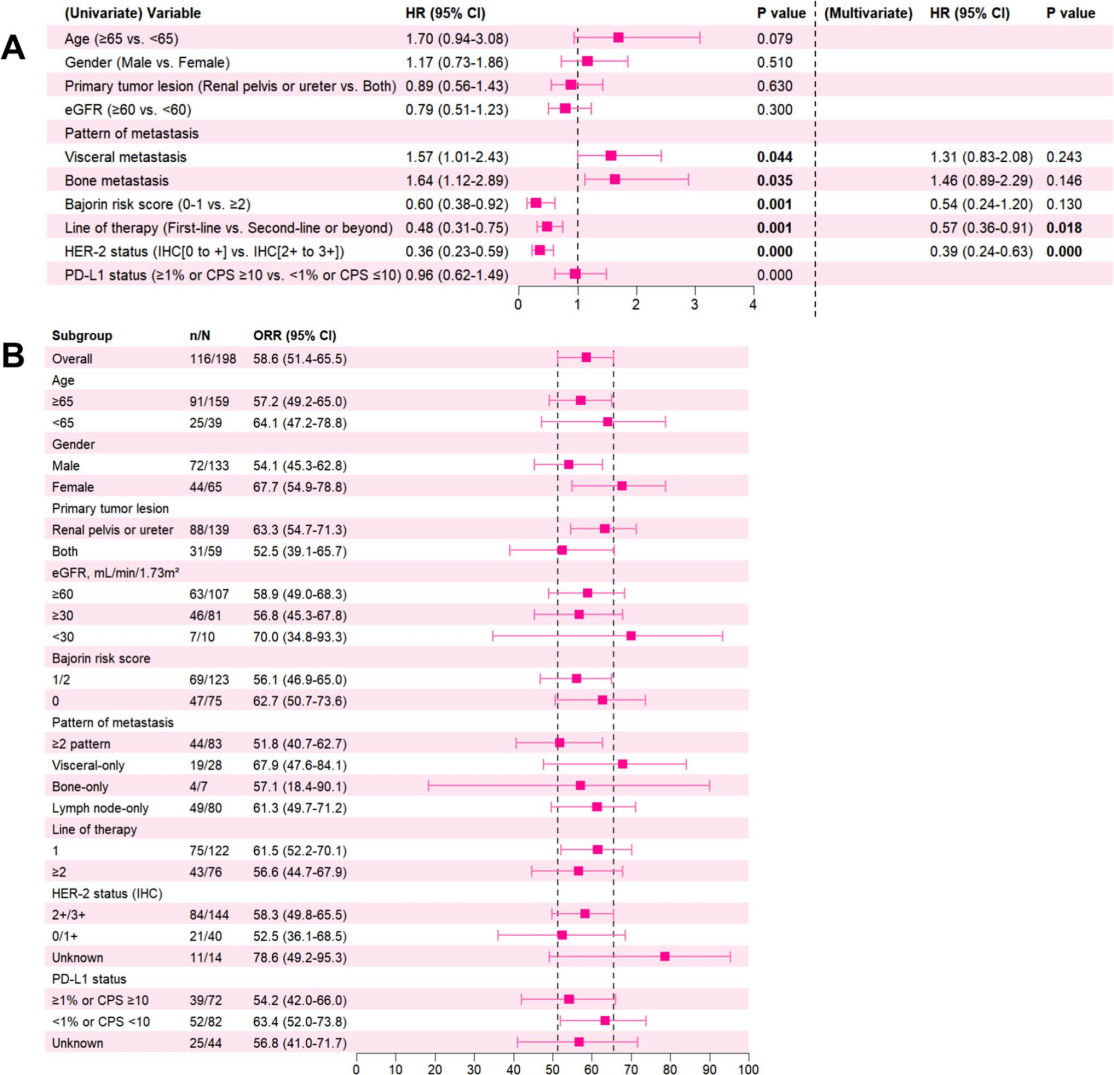
Comparison of PFS in different patient subgroups by univariate and multivariate cox regression analysis **(A)** and comparison of ORR across patient subgroups **(B)**

In the cohort of patients treated with RC48-ADC plus ICI, the ORR was 58.6% (95%CI: 51.4–65.5, 116 of 198 patients), of which 16 (8.1%) patients had a CR and 100 (50.5%) patients achieved PR. The median time to response was 3.0 months (IQR: 2.0–4.0 months). The median duration of response (DoR) had not been reached (95%CI: 11.0-NA) (Fig. 1A). The results of survival analysis and corresponding survival curves are presented in Fig. 1B–D. With a median follow-up time of 13.0 months (95%CI: 12.4–13.5), PFS events were observed in 82 (41.4%) patients. The median PFS (mPFS) was 13.0 months (95%CI: 10.0-NA), with estimated 12-month and 18-month PFS rates of 51.3% and 45.4%, respectively. OS events were observed in 17 (8.6%) patients, with the median OS not reached, and the estimate 12-month and 18-month OS rates of 90.8% and 84.2%, respectively (Fig. 1B). Subgroup survival curves demonstrated that HER-2 status, line of treatment, visceral, and bone metastasis status were significant factors affecting patients’ PFS duration (Fig. 2). Patients presented with HER-2 IHC scores of 2 + or 3 + had significantly longer mPFS compared with patients with HER-2 scores of 0 or 1 + (NA vs. 8 months, *P* < 0.001), while no significant differences were observed in patients with varying PD-L1 expression (Fig. 2A and B). Similarly, significant differences in PFS were observed across subgroups stratified by treatment line and baseline metastatic status (visceral or bone involvement), as demonstrated in Figs. 2C, D, and E. Notably, all patients who achieved a CR as their best overall response did not experience PD or death. S- Fig. 2 shows the survival curves of patients across different subgroups, and longer OS was observed in subgroups of bone metastasis status and line of therapy. No survival difference was observed in patients grouped by eGFR (Fig. 2E and S- Fig. 2E). Furthermore, no significant differences in survival outcomes were observed among patients receiving different ICIs (S- Fig. 3).

As shown in Fig. 3, significant PFS benefits were observed at subgroups of line of therapy (first-line vs. second line or beyond, *P* = 0.013) and HER-2 expression [IHC (2 + or 3 +) vs. IHC (0 or 1 +), *P* = 0.024]. The PFS observed in other prespecified subgroups (such as age, gender, primary tumor location, eGFR, and PD-L1 expression) was consistent (Fig. 3A). The ORR was consistent across all subgroups and aligned with the overall ORR (Fig. 3B). In the subgroup of patients with HER-2-positive disease (IHC 2 + or 3 +) receiving first-line combination therapy, median PFS was not reached, and ORR was 59.4%.

### Safety

Out of the 198 patients treated with combination therapy, 158 (79.8%) patients experienced at least one AE of any grade. The most common AEs were peripheral sensory neuropathy (40.4%), fatigue (26.8%), alopecia (25.3%), and rash (21.7%). Grade 3–4 AE occurred in 19.7% of patients, the most common grade 3 or higher events were peripheral sensory neuropathy (9.1%), rash (4.0%), and leukopenia (2.5%). The detailed record of AEs is listed in Table 2. No patients died due to serious AEs. Dose reductions due to AEs occurred in 13 (6.6%) patients, and complete discontinuation of either RC48-ADC and/or ICIs due to AEs occurred in 8.6% of patients **(**S- Table 1).

**Table 2:** Treatment-related adverse events (TRAEs) of patients

Adverse event	Any grade	Grade ≥ 3
All patients	158 (79.8%)	39 (19.7%)
Peripheral sensory neuropathy (sensory loss, numbness in hands and feet)	80 (40.4%)	18 (9.1%)
Fatigue	53 (26.8%)	4 (2.0%)
Alopecia	50 (25.3%)	0
Rash	43(21.7%)	8 (4.0%)
Pruritus	34 (17.2%)	4 (2.0%)
Increased ALT	32 (16.2%)	4 (2.0%)
Increased AST	24 (12.1%)	4 (2.0%)
Leukopenia	24 (12.1%)	5 (2.5%)
Decrease in appetite	15 (7.6%)	0
Increased serum creatinine	13 (6.6%)	3 (1.4%)
Diarrhea	12 (6.1%)	1 (0.5%)
Anemia	11 (5.6%)	3 (1.4%)
Nausea	11 (5.6%)	0
Cough	11 (5.6%)	0
Hypothyroidism	9 (4.5%)	1 (0.5%)
Neutropenia	9 (4.5%)	1 (0.5%)
Vomiting	7 (3.5%)	0
Thrombocytopenia	4 (2.0%)	4 (2.0%)
Edema	4 (2.0%)	1 (0.5%)
Fever	4 (2.0%)	1 (0.5%)
Constipation	4 (2.0%)	0
Hypertension	4 (2.0%)	0
Lower back pain	4 (2.0%)	0
Increased triglycerides	3 (1.5%)	0
Oral mucositis	3 (1.5%)	0
Xerostomia	3 (1.5%)	0
Dyspnea	2 (1.0%)	1 (0.5%)
Increased creatine kinase	2 (1.0%)	1 (0.5%)
Arthralgia	2 (1.0%)	0
Immune-mediated interstitial pneumonia	1 (0.5%)	1 (0.5%)
Intestinal obstruction	1 (0.5%)	1 (0.5%)
Altered consciousness	1 (0.5%)	1 (0.5%)
Elevated troponin	1 (0.5%)	1 (0.5%)
Pericardial effusion	1 (0.5%)	1 (0.5%)
Blurred vision	1 (0.5%)	0
Cholecystitis	1 (0.5%)	0
Gastritis	1 (0.5%)	0
Generalized pain	1 (0.5%)	0
Hematuria	1 (0.5%)	0
Hoarseness	1 (0.5%)	0
Hyperglycemia	1 (0.5%)	0
Stomatitis	1 (0.5%)	0
Urinary tract infection	1 (0.5%)	0

## Discussion

To the best of our knowledge, this study represents the first and the largest retrospective study evaluating the efficacy and safety of RC48-ADC in combination with ICIs in patients with mUTUC, even in the broader context of mUC. In this multicenter, retrospective real-world study, the RC48-ADC in combination with ICIs showed promising efficacy and manageable safety in patients with mUTUC, with the ORR of 58.6% and mPFS of 13.0 months, the 12-month PFS rate and OS rate were 47.2% and 89.6%, respectively. Most of the AEs could be managed. Notably, for patients with decreased eGFR at baseline (< 60 ml/min/1.73m^2^), the combination therapy also showed good efficacy and safety. The findings of this study may provide evidence for the clinical benefit of RC48-ADC plus ICIs in the treatment of mUTUC. In addition, the combination therapy may offer a new treatment option for cisplatin-ineligible patients.

In the first-line treatment of metastatic upper tract urothelial carcinoma (mUTUC), cisplatin-based chemotherapy remains the standard, offering a median progression-free survival (mPFS) of 8 months and overall survival (mOS) of 15 months [12, 13]. However, impaired renal function in many UTUC patients limits its use, making immune checkpoint inhibitors (ICIs) a viable alternative. The IMvigor130 trial showed atezolizumab monotherapy achieved an mPFS of 6.3 months and mOS of 15.7 months, while the KEYNOTE-052 trial reported pembrolizumab had an overall response rate (ORR) of 28.6%, mOS of 11.3 months, and mPFS of 2.2 months in cisplatin-ineligible patients [14, 15]. In our study, combination therapy with RC48-ADC and ICIs in a cisplatin-ineligible subgroup yielded an ORR of 61.5%, with 12-month progression-free and overall survival rates of 60.8% and 92.9%, respectively. Despite the different characteristics of the study population, our results demonstrated a considerable effectiveness compared to the aforementioned studies. Further studies are needed to explore the efficacy of combination therapy of RC48-ADC and ICIs.

Approximately 50% of UC patients showed HER-2 positivity (IHC 2 + or 3 +) [16, 17]. However, the anti-HER-2 agents such as HER-2 tyrosine kinase inhibitors (HER-2-TKIs) showed Limited efficacy in mUC. For instance, the overall response rate was 8.7% in patients with metastatic platinum-refractory UC using Afatinib [18]. In contract, ADCs targeting HER-2 showed promising results. The RC48-C005 and RC48-C009 trials demonstrated the consistently promising efficacy of RC48-ADC in patients with HER-2 positive, chemotherapy-refractory mUC, with an overall ORR of 50.5%, a PFS of 5.9 months, and an OS of 14.2 months [10]. Besides, for patients with low HER-2 expression, the RC48-C011 study also indicated certain antitumor activity of RC48-ADC in HER-2 negative metastatic UC, with a confirmed ORR of 26.3%, a mPFS of 5.6 months, and a mOS of 16.4 months [19]. However, challenges such as payload toxicities, antigen heterogeneity, and adaptive resistance contributing to ADC drug resistance still need to be addressed [20].

Several ADCs, including RC48-ADC, enfortumab vedotin (EV), and sacituzumab govitecan (SG), have shown success in chemotherapy-refractory mUC. The EV-302 trial reported an ORR of 67.7%, mPFS of 12.5 months, and mOS of 31.5 months for EV plus pembrolizumab as first-line therapy [21]. In the TROPHY-U-01 trial, SG combined with pembrolizumab as second-line therapy achieved an ORR of 41%, mPFS of 5.3 months, and mOS of 12.7 months, though further exploration is needed for its first-line use [22]. In our study of RC48-ADC plus ICIs, the mPFS was 13.0 months, and mOS was not reached. Patients with HER-2 IHC (2 + or 3 +) receiving first-line therapy had significantly better outcomes (mPFS was not reached, and ORR was 59.4%), suggesting that HER-2 status and treatment line influence efficacy. This combination also appears more effective than RC48-ADC monotherapy, likely due to synergy between ADCs and immunotherapy, which may overcome resistance and amplify immune responses [23–25]. The RC48-014 trial, testing DV with toripalimab in mUC regardless of HER-2 status, has shown an ORR of 73.2%, including 9.8% CR, with a median PFS of 9.3 months and median OS of 33.1 months [26]. These results underline the need for further large-cohort prospective studies to validate these findings and clarify mechanisms enhancing clinical efficacy.

Compared to bladder cancer (BC), UTUC is often considered to exhibit a higher propensity for metastasis [27]. Although direct comparisons cannot be made due to differences in inclusion criteria and potential statistical bias inherent to retrospective studies, the survival result of this study, which included only patients with UTUC, share similarities with those reported in other studies on mUC which may improve the relatively poor prognosis of UTUC. Previous studies involving other ADCs in combination with ICIs also indicated that no statistical differences were observed between the survival of UTUC patients and BC patients [21, 22, 28].

Our study indicated that the toxicity of RC48-ADC combined with ICIs was clinically manageable. The most common AEs included peripheral sensory neuropathy, fatigue, alopecia, and rash, which could be alleviated or controlled through symptomatic treatments. A minority of patients presented with serious AEs (grade 4), such as pericardial effusion, altered consciousness, and intestinal obstruction. Only 8.6% of patients discontinued treatment of ADCs and/or ICIs. The AEs reported at our study are similar to findings reported in previous studies of RC48-ADCs and ICIs [10, 29]. When comparing with other ADCs-related studies, the AEs of RC48-ADC plus ICIs are consistent—other studies utilizing ADC-immunotherapy—with neuropathy, alopecia and fatigue being more common. However, the synergy between ADCs and ICIs is not clearly studied, such as neurotoxicity; the rate was similar when comparing DV plus ICIs with DV monotherapy (63.4% in combination vs. 68.2% in DV monotherapy), while EV plus pembrolizumab showed higher rate of peripheral sensory neuropathy (50.0% in combination vs. 34.8% in EV monotherapy) [10, 21, 26, 30].

While this analysis provides novel real-world evidence of therapeutic activity in mUTUC—a population with critically limited treatment options—several limitations warrant acknowledgment. The retrospective design limited comprehensive balancing of baseline characteristics and systematic collection of key prognostic variables including comorbidity profiles and secondary malignancy histories. Exclusion of patients with insufficient treatment duration (*n* = 35) may also introduce selection bias by excluding patients with early progression or toxicity, although this reflects real-world evaluability criteria. Furthermore, the absence of a comparator cohort precludes definitive causal inferences regarding treatment efficacy. Nevertheless, these findings deliver unique clinical insights for this high-need population, with the observed objective response rate of 58.6% and median progression-free survival of 13.0 months offering clinically actionable benchmarks pending validation in randomized trials. Importantly, the sole inclusion of Chinese patients introduces geographical bias, as Asian and Western upper tract urothelial carcinoma (UTUC) populations demonstrate significant epidemiological divergence; UTUC accounts for 20–30% of urothelial carcinomas in Asian countries versus 5–10% in Western populations, with lower prior bladder cancer rates (4% vs. 41%) yet higher prevalence of adverse features including high-grade pathology (98% vs. 77%), muscle-invasive disease (≥ pT2 stage: 64% vs. 38%), and preoperative hydronephrosis (56% vs. 40%) [31]. Molecular profiling further revealed elevated FGFR3 mutation rates (45% vs. 32%) and increased HER-2-high expression (IHC 2 + /3 + : 44.0% in Chinese cohorts vs. 35.8% in Western counterparts) [31–33], potentially modulating combination therapy efficacy. Critically, China’s medical insurance coverage for RC48-ADC—restricted to HER-2-high patients—introduces inherent therapeutic selection bias, enriching this responsive subgroup and potentially overestimating overall treatment benefits. These collective limitations fundamentally restrict the generalizability of findings across broader demographics. Consequently, multinational validation studies in ethnically diverse cohorts are imperative to confirm RC48-ADC/ICI efficacy and evaluate biomarker dependency.

## Conclusions

In summary, this real-world study demonstrated the efficacy of combination therapy of RC48-ADC plus ICIs in mUTUC patients. Furthermore, this combination did not lead to a significant increase in adverse events, with the safety profile consistent with previous studies about RC48-ADC and ICIs. Therefore, it holds promise as a new therapeutic option for mUTUC, particularly in pre-selected patient populations.

## Supplementary Information

Below is the link to the electronic supplementary material.

Supplementary file1 (DOCX 974 KB)

## Data Availability

The datasets generated and/or analyzed during the current study are available from the corresponding author upon reasonable request.

## Abbreviations

AE: Adverse events
EV: Enfortumab vedotin
DV, RC48-ADC: Disitamab vedotin
ICI: Immune checkpoint inhibitor
mUC: Metastatic urothelial carcinoma
mUTUC: Metastatic upper tract urothelial carcinoma
ORR: Objective response rate
OS: Overall survival
PFS: Progression-free survival
SG: Sacituzumab govitecan
TRAE: Treatment-related adverse event
