# Supplementary material for: The efficacy and safety of disitamab vedotin combined with immune checkpoint inhibitors in metastatic upper tract urothelial carcinoma: a multicenter real-world study
Source: Cancer Immunol Immunother. 2025 Sep 13;74(10):304. doi: 10.1007/s00262-025-04154-5 (PMC12433416; doi:10.1007/s00262-025-04154-5)

**Supplemental materials**

| S-Table 1 Medication changes due to adverse events | | | | |
| --- | --- | --- | --- | --- |
| **Medication changes due to adverse events** | **Total** | **RC48-ADC** | **ICI** | **Both** |
| No reduction or discontinuation | 138 (69.7%) | - | - | - |
| Leading to dose reduction | 13 (6.6%) | 13 (6.6%) | 0 | 0 |
| Leading to drug discontinuation | 47 (23.7%) | 27 (13.6%) | 4 (2.0%) | 16 (8.1%) |
| Leading to complete discontinuation of drug(s) | 17 (8.6%) | - | - | - |

**S-figure 1** Flow chart of the study


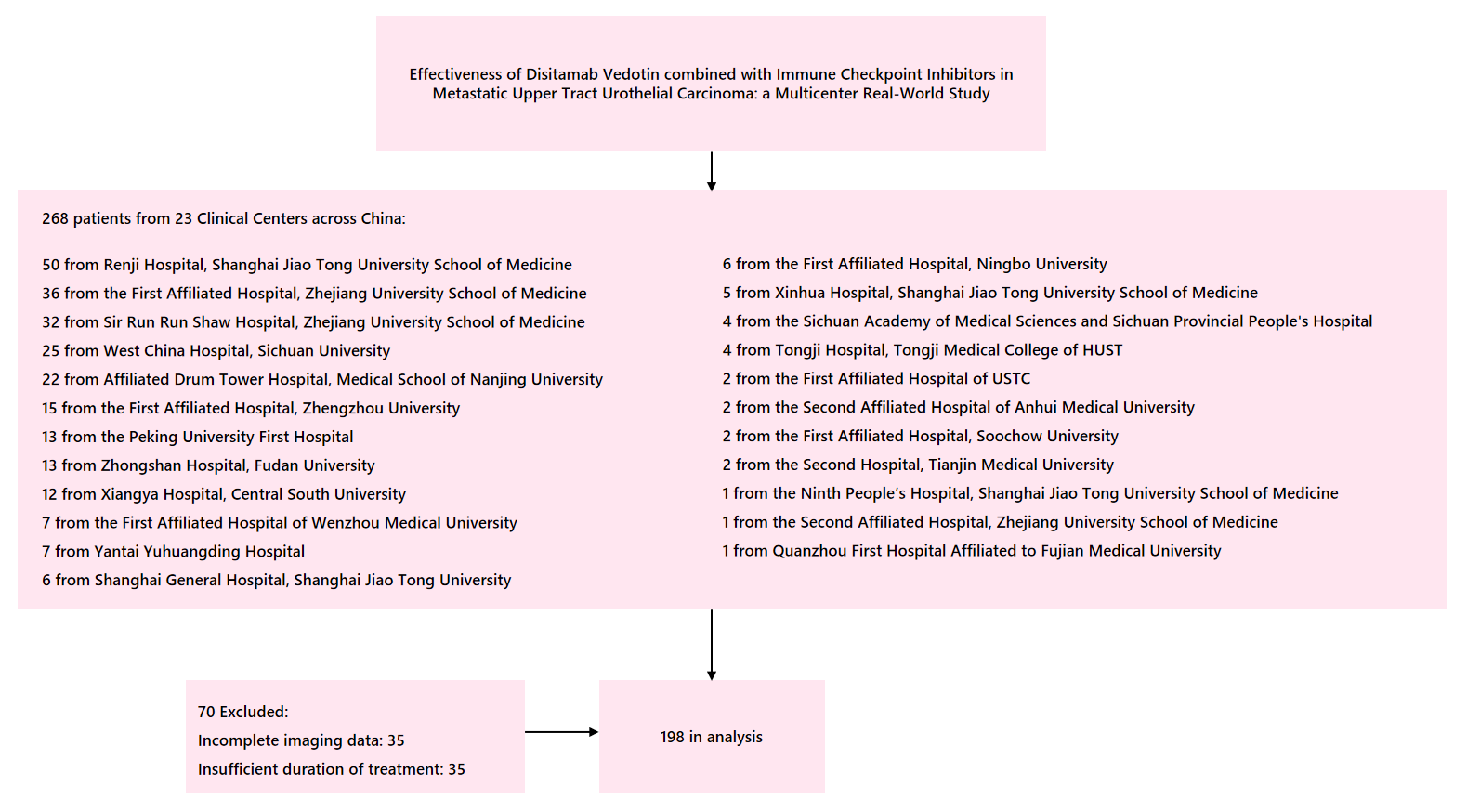


**S-figure 2** Overall survival of patients in different subgroups


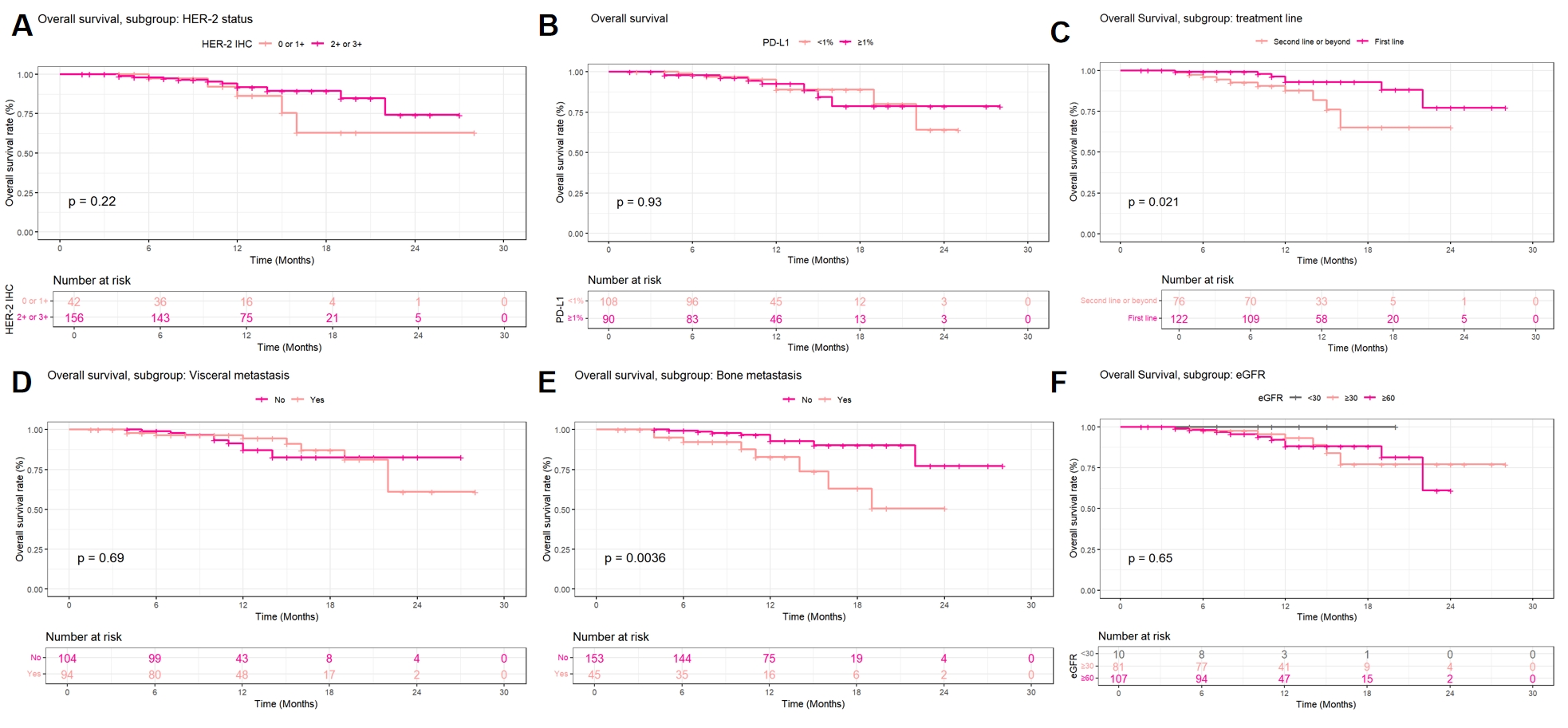


1. **figure 3** Survival analysis of patients underwent different ICIs. (A)Progression-free survival; (B) Overall survival; (C) P value of different subgroups’ progression-free survival.


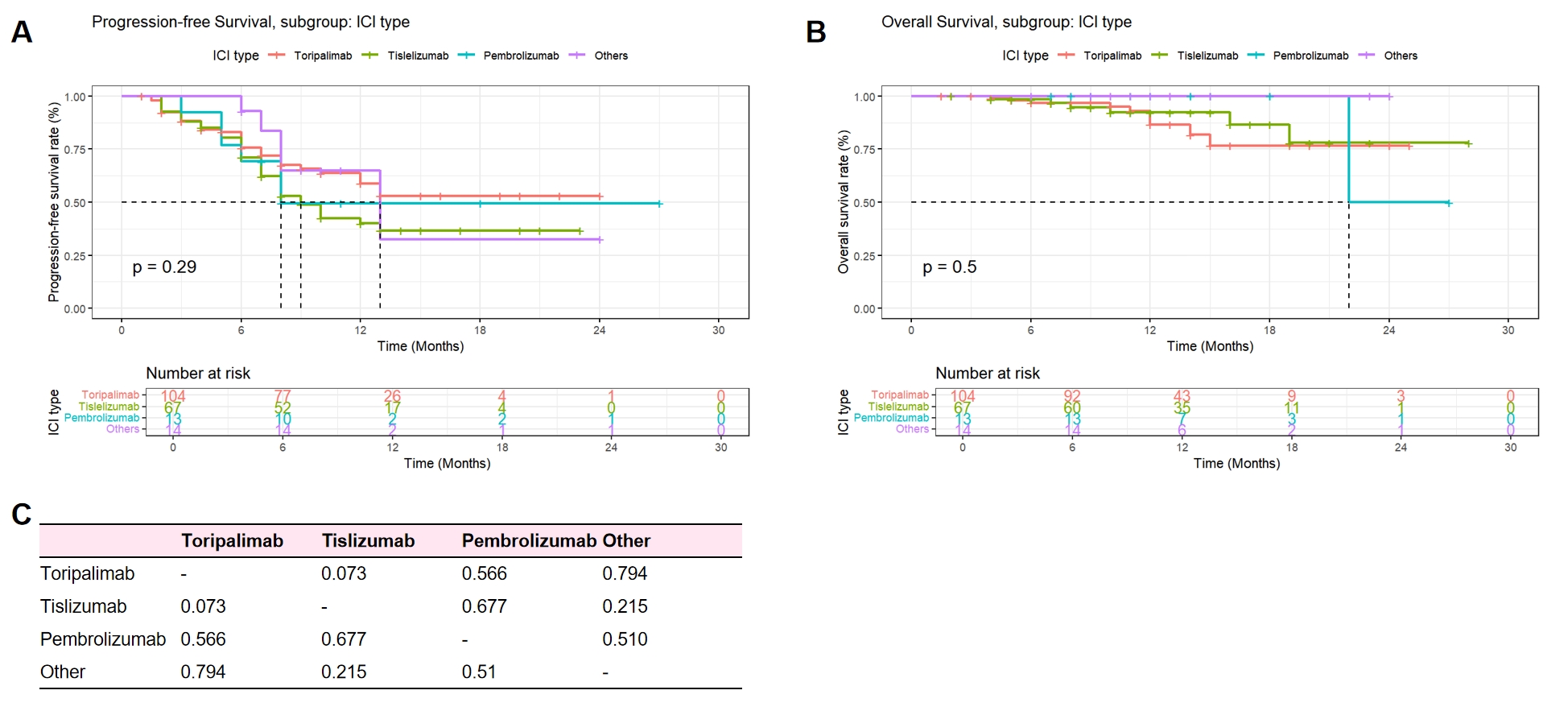

Supplement: Supplementary file 1 — Supplementary file1 (DOCX 974 KB) [file 262_2025_4154_MOESM1_ESM.docx]
